# Stable Cholesterol–Palmitic Acid Sterosomes as Smart Nanocarriers for pH-Sensitive Doxorubicin Delivery in Breast Cancer Therapy

**DOI:** 10.3390/pharmaceutics17121574

**Published:** 2025-12-06

**Authors:** Jeong Min Lee, Chung-Sung Lee, Chae Yeong Lee, Min Lee, Hee Sook Hwang

**Affiliations:** 1 Department of Pharmaceutical Engineering, Dankook University, Cheonan 31116, Republic of Korea; 2 Department of Pharmaceutical Engineering, Soonchunhyang University, Asan 31538, Republic of Korea; chungsung@sch.ac.kr; 3 Institute for Molecular Metabolism Innovation, Soonchunhyang University, Asan 31538, Republic of Korea; 4 Division of Oral and Systemic Health Sciences, School of Dentistry, University of California, Los Angeles, CA 90095, USA; 5 Department of Bioengineering, University of California, Los Angeles, CA 90095, USA

**Keywords:** sterosome, non-phospholipid nanoparticle, breast cancer therapy, doxorubicin, pH sensitive drug release

## Abstract

**Background**: Breast cancer remains one of the most prevalent and lethal malignancies worldwide. Although doxorubicin (DOX) is widely used as a first-line chemotherapeutic agent, its clinical utility is constrained by dose-limiting cardiotoxicity and systemic adverse effects. Nanoparticulate drug delivery systems have therefore attracted attention for improving DOX stability, biocompatibility, and tumor selectivity. In this study, we explored sterosomes—simple non-phospholipid nanocarriers composed of cholesterol and palmitic acid—as an alternative DOX delivery platform with pH-responsive properties. **Methods**: DOX-loaded sterosomes (DOX-STs) were prepared using cholesterol and palmitic acid to impart acid-sensitive behavior. The nanocarriers were systematically evaluated through particle characterization, physicochemical stability assessment, in vitro pH-dependent drug release, and cellular uptake studies. Furthermore, therapeutic efficacy and systemic safety were investigated in an MDA-MB-231 breast cancer xenograft mouse model. **Results**: DOX-STs exhibited particle sizes below 100 nm, high encapsulation efficiency, and excellent colloidal stability for 28 days. The sterosomes demonstrated accelerated DOX release under acidic conditions relative to physiological pH, consistent with their pH-responsive design. Enhanced cellular uptake was observed in both MCF-7 and MDA-MB-231 cells. In vivo, DOX-ST treatment resulted in significant tumor growth suppression and prolonged survival without notable body weight loss, indicating reduced systemic toxicity compared to free DOX. **Conclusions**: This study presents a simple sterosome-based nanocarrier system that achieves pH-responsive DOX release and enhanced antitumor efficacy while minimizing toxicity. These findings highlight the potential of sterosomes as a translatable nanomedicine platform for breast cancer therapy.

## 1. Introduction

Breast cancer is among the most frequently diagnosed cancers globally, accounting for approximately 11.7% of all cases in 2020 [1]. Breast cancer represents a significant malignancy and is the second leading cause of cancer-related mortality in women, primarily due to metastasis resulting from rapid cellular differentiation, decreased apoptosis, and tumor-associated angiogenesis [2,3]. Hormone-receptor-positive (HR+) breast cancer, which constitutes the majority of breast cancer cases, has great potential for targeted therapy owing to the presence of specific biomarkers [4]. In contrast, triple-negative breast cancer (TNBC), which is characterized by the absence of estrogen, progesterone, and human epidermal growth factor receptor 2 (HER2) expression, has limited options for targeted therapies. TNBC correlates with a poor prognosis, as it is frequently associated with a high recurrence rate, aggressive tumor growth, and rapid mitotic activity [4,5,6,7,8,9]. The principal treatment approaches for breast cancer comprise surgery, chemotherapy, and radiation therapy [10,11].

Among chemotherapeutic agents, doxorubicin (DOX) is extensively used in the treatment of breast cancer [12]. As an anthracycline antibiotic, DOX acts as a cell-cycle-nonspecific agent and is commonly administered in chemotherapy regimens for both ectopic and metastatic breast cancer [5,13]. Its mechanism of anticancer activity centers on the inhibition of topoisomerase I and II, resulting in DNA damage and subsequent induction of apoptosis in cancer cells [13,14]. In addition, DOX intercalates into DNA through its anthracycline ring structure, causing direct structural disruption and generating reactive oxygen species (ROS). These events contribute to damage and death not only in cancer cells but also in normal cells [13,15]. Despite its efficacy, its clinical use is limited by various toxicities, most notably cardiotoxicity [16].

To address these limitations, research has increasingly focused on nanoparticulate drug delivery systems. Liposomes and polymeric nanoparticles are among the most investigated platforms. Liposomes are vesicular carriers formed by dispersing cholesterol (Chol) and phospholipids in aqueous environments. Their resemblance to the cell membrane improves drug stability and enables targeted drug delivery to tumor sites, which in turn reduces nonspecific systemic exposure and decreases adverse effects on healthy tissues [17,18,19,20]. Nonetheless, liposomes primarily composed of phospholipids can be inherently unstable, posing challenges for prolonged storage. Moreover, the encapsulation efficiency may be suboptimal for certain drugs, requiring an increase in drug dosage. For hydrophilic drugs, premature release from the aqueous core of liposomes can occur, potentially impeding the achievement of sustained drug delivery [21,22].

Polymeric nanoparticles such as PLGA have been widely explored due to their biodegradability, regulatory approval, and ability to provide controlled and sustained drug release [23,24,25,26,27]. However, unmodified PLGA nanoparticles generally exhibit limited cellular uptake and require multistep, high-cost manufacturing processes, which has motivated the search for simpler and more tunable delivery platforms [28,29,30,31]. In a recent study, Chronopoulou et al. reported DOX-loaded PLGA particles displaying exceptionally high loading efficiency (up to ~99%) and notable cytotoxicity in MCF-7 cells, primarily through plasma-membrane adhesion and reservoir-type sustained DOX release rather than intracellular nanoparticle uptake [32]. While effective, this system demonstrated an initial burst release and lacked intrinsic pH-responsive behavior, underscoring the need for nanocarriers that can achieve controlled release while offering simpler fabrication and improved physicochemical stability.

To address these limitations, sterosomes—a liposome-inspired nanocarrier platform composed of non-phospholipids and high sterol content—have emerged as a promising alternative [18]. Sterosomes self-assemble into stable, fluid bilayers and possess enhanced structural robustness compared with conventional phospholipid-based liposomes [33]. Furthermore, sterosomes can be engineered to provide enhanced structural stability over conventional phospholipid-based liposomes and exhibit diverse functional attributes, such as pH sensitivity, antibacterial activity, and suitability for bone regeneration and remodeling applications [34,35]. Their physicochemical characteristics confer long-term colloidal stability, low membrane permeability, and extended in vivo circulation and retention [33,36,37]. Moreover, sterosomes inherently exhibit pH-responsive behavior and can be fabricated through a simple thin-film hydration method with lower production costs and greater storage stability than both phospholipid liposomes and PLGA nanoparticles [33,38]. Owing to these advantages, sterosomes have been successfully utilized for the delivery of small-molecule drugs, nucleic acids, and other therapeutic agents, positioning them as a versatile and cost-effective platform for anticancer drug delivery.

In this study, sterosomes were formulated using Chol and palmitic acid (PA). Chol, an essential constituent of sterosomes, is recognized for its ability to modulate membrane fluidity and permeability. Studies have indicated that it increases the stability of bilayer membranes in biological fluids such as blood [39,40]. Additionally, Chol has been demonstrated to decrease membrane permeability to water-soluble molecules [41]. PA, another crucial sterosome component, is a long-chain saturated free fatty acid that serves as a regulator for membrane permeability and associated functions [42]. Moreover, PA contributes to maintaining bilayer fluidity by occupying spaces between Chol molecules [43]. The incorporation of PA allows the nanoparticles to acquire pH sensitivity [44]. In addition, sterosomes comprising Chol and PA have been shown to exhibit greater chemical stability and reduced bilayer permeability compared to alternative sterosome formulations in a previous study [45]. On the basis of these properties, Chol and PA were chosen as the main constituents for sterosome synthesis in this study.

Sterosomes were synthesized using a lipid film method [22]. The protonation state of PA in Chol/PA-based sterosomes has been shown to affect bilayer stability, leading to pH-responsive behavior in a previous study [24]. These findings indicate that the sterosomes produced in this investigation may be capable of facilitating pH-responsive drug release. Subsequently, DOX, classified as a cell-cycle-nonspecific chemotherapeutic agent, was incorporated into the vesicles. In this study, we aim to systematically investigate Chol/PA-based sterosomes as pH-responsive nanocarriers for doxorubicin by characterizing their physicochemical properties, drug-loading capacity, pH-dependent release behavior, and evaluating their potential applicability in breast cancer therapy through comprehensive in vitro and in vivo studies.

## 2. Materials and Methods

### 2.1. Materials

Doxorubicin hydrochloride (DOX∙HCl) was purchased from Aladdin (Meridian, CA, USA). Chol and 3-(4,5-dimethyl-2-thiazolyl)-2,5-diphenyl-2H-tetrazolium bromide (MTT) were obtained from Tokyo Chemical Industry Co. (Saitama, Japan). Absolute ethanol (99.9%) was purchased from Samchun (Pyeongtaek, Korea). Phosphate-buffered saline (PBS, 0.01 M) was obtained from Dyne Bio (Seongnam, Korea). Dichloromethane and PA were purchased from Daejung (Siheung, Korea). Dimethyl sulfoxide (DMSO) and polyoxyethylene-20-sorbitan monooleate (Tween 80) were purchased from HanLab (Cheongju, Korea). Triethylamine (TEA) was obtained from Sigma-Aldrich Co. (St. Louis, MO, USA). Dialysis tubing (molecular weight cut-off 3.5 kDa) was purchased from AS ONE (Osaka, Japan). Dulbecco’s modified Eagle’s medium (DMEM) and fetal bovine serum (FBS) were obtained from Welgene (Daegu, Korea). Antibiotic/antimycotic solution was purchased from Cytiva (Washington, DC, USA). Trypsin-EDTA solution (0.25%, 1×) was obtained from Gibco (Invitrogen Corp., Carlsbad, CA, USA). Hoechst 33342 was purchased from Thermo Fisher Scientific (Waltham, MA, USA).

### 2.2. Preparation of Sterosomes

Sterosomes were fabricated via the thin-film hydration technique [22]. Chol and PA were each dissolved in ethanol under vigorous stirring at room temperature, mixed at a 1:1 molar ratio, and transferred into a round-bottom flask. The solvent was removed at 37 °C using a rotary evaporator, resulting in a thin lipid film that was subsequently hydrated with PBS (0.01 M, pH 7.4) and subjected to sonication with a tip sonicator (20 s on/5 s off, 20% amplitude, 25 W/cm^2^) to yield blank sterosomes (Blank-ST). To generate desalted DOX-loaded sterosomes (DOX-ST), Chol, PA, and desalted DOX were combined in a 1:1:0.1 molar ratio, dissolved in ethanol, and processed using the same thin-film hydration protocol. After hydration with PBS (0.01 M, pH 7.4) and identical sonication, the resulting dispersion was filtered through a 0.45 μm syringe filter to eliminate free DOX. This 1:1:0.1 molar ratio (Chol:PA:DOX) was selected based on preliminary optimization studies, which showed that increasing the DOX proportion above 0.1 molar equivalents did not further improve encapsulation efficiency, indicating saturation of the sterosome loading capacity under these formulation conditions.

### 2.3. Characterization of Sterosomes

Dynamic light scattering (DLS; Zetasizer Pro, Malvern Instruments Ltd., Malvern, UK) was employed to assess the mean diameter, polydispersity index (PDI), and zeta potential of the sterosomes at 25 °C. Prior to analysis, all formulations were passed through 0.22 μm filters and diluted tenfold with distilled water. All DLS and zeta potential measurements were performed at pH 7.4 unless otherwise specified. All measurements were obtained in triplicate. The morphologies of sterosomes were examined using transmission electron microscopy (TEM; JEM-2100F, JEOL Ltd., Tokyo, Japan).

### 2.4. Determination of DOX Content in Sterosomes

The quantity of DOX incorporated into sterosomes was evaluated using a microplate reader (Synergy HTX, Agilent, Santa Clara, CA, USA). After removing free DOX by filtration through a 0.45 μm membrane, the sterosome membranes were disrupted with 0.05% dichloromethane to release the encapsulated DOX. The absorbance of the released (encapsulated) DOX was then measured at 480 nm utilizing the microplate reader. Encapsulation efficiency (EE) and drug loading content (DLC) were computed according to Equations (1) and (2), respectively. EE and DLC were evaluated under physiological pH conditions (pH 7.4). All assays were conducted in triplicate. Preliminary variation in the DOX amount (0.05–0.2 molar equivalents) showed no improvement in EE beyond the 0.1 equivalent used in the optimized formulation.

(1) $$\mathrm{EE}\;(\%)=\frac{{\left[DOX\right]}_{encapsulated\;in\;sterosome}}{{\left[DOX\right]}_{total}}\times 100$$

(2) $$\mathrm{DLC}\;(\%)=\frac{Weight\;of\;DOX}{Weight\;of\;sterosome}\times 100$$

### 2.5. Sterosomes Stability

DOX-STs stability was investigated by storing samples at 4 °C for 28 days. Measurements of particle size, PDI, and zeta potential were collected at specified time points over the 28 days using dynamic light scattering (DLS; Zetasizer Pro, Malvern Instruments Ltd., UK). Particle stability was monitored at pH 7.4 during the entire testing period. Additionally, the EE of DOX-STs was assessed at each interval for 28 days. EE was determined using a microplate reader (Synergy HTX, Agilent, Santa Clara, CA, USA) and calculated by Equation (1). To additionally evaluate serum stability, DOX-STs were incubated in PBS (0.01 M) containing 10% fetal bovine serum (FBS) at 37 °C for 24 h. Following incubation, particle size, PDI, zeta potential, and EE were measured using the same analytical procedures described above.

### 2.6. In Vitro Drug Release

The in vitro release of DOX-STs was assessed using the dialysis method at 37 °C under gentle continuous agitation. In summary, 10 mL of the DOX-STs suspension was placed in a dialysis bag and immersed in 90 mL of PBS (0.01 M, pH 7.4, 6.5, or 5.5) containing 1% Tween 80. At specified time points over a total duration of 96 h, 1 mL of the release medium was withdrawn and stored at 4 °C for subsequent analysis, and the same volume of fresh buffer was added to maintain sink conditions. The collected samples were transferred into a 96-well plate, and DOX concentration was quantified using a microplate reader (Synergy HTX, Agilent, Santa Clara, CA, USA) at 480 nm. Each experiment was conducted in triplicate.

### 2.7. Cell Culture and Incubation Conditions

MDA-MB-231 (human breast cancer, KCLB No. 30026), HepG2 (human liver cancer, KCLB No. 88065), A549 (human lung cancer, KCLB No. 10185), MCF-7 (human breast cancer, KCLB No. 30022), and NIH3T3 (mouse embryonic fibroblast, KCLB No. 21658) cell lines were obtained from the Korean Cell Line Bank (Seoul, Korea). These cells were cultured in DMEM supplemented with 10% FBS and 1% antibiotic/antimycotic solution. All cultures were incubated at 37 °C in a 5% CO_2_ humidified environment and sub-cultured every 2–3 days using fresh medium. For all experiments, cells between passages 5 and 10 were used to ensure consistency.

### 2.8. In Vitro Cell Viability Test

The cytotoxicity of DOX-STs was assessed in MDA-MB-231, HepG2, A549, MCF-7, and NIH3T3 cell lines by performing the MTT assay. In brief, cells were seeded into 96-well plates at a density of 5 × 10^3^ cells per well and incubated at 37 °C in a humidified atmosphere with 5% CO_2_ for 24 h. The same seeding density was applied to all cell lines. The cells were subsequently exposed to varying concentrations (0–10 μM) of either free DOX or DOX-STs for an additional 24 h under the same conditions. Afterward, 25 μL of MTT solution (0.5 mg/mL) was added to each well and incubated for 4 h. The medium was then aspirated, and 100 μL of DMSO was introduced to dissolve the formazan crystals, followed by a 30 min incubation. Absorbance was recorded at 570 nm with a microplate reader (Synergy HTX, Agilent, Santa Clara, CA, USA). Cell viability was calculated according to Equation (3). All experiments were conducted in triplicate. IC_50_ values were calculated using nonlinear regression analysis (log(inhibitor) vs. response, variable slope) in GraphPad Prism (version 8.0.1, GraphPad Software, San Diego, CA, USA).

(3) $$\mathrm{Cell}\;\mathrm{viability}\;(\%)=\frac{Sample’s\;Absorbance}{Control’s\;Absorbance}\times 100$$

### 2.9. In Vitro Cellular Uptake

The cellular uptake of DOX-STs and free DOX was assessed in MCF-7 and MDA-MB-231 cells. Cells were seeded in 6-well plates at a density of 3 × 10^5^ cells/well and incubated at 37 °C in a humidified 5% CO_2_ atmosphere for 24 h. The same seeding density was applied to all cell lines. Following incubation, the medium was replaced with fresh medium containing either free DOX or DOX-STs at a final concentration of 5 μM, and cells were further incubated for 4, 6, or 12 h under identical conditions. At specified time points, the medium was aspirated, and cells were washed with PBS (0.01 M, pH 7.4). Nuclei staining was performed using Hoechst 33342 (100 μL) for 30 min, after which the staining solution was removed and the cells were rinsed again with PBS (0.01 M, pH 7.4). The cells were then fixed, and uptake of intracellular DOX was observed using a fluorescence microscope (Nikon, Tokyo, Japan). Quantification of fluorescence intensity was performed using ImageJ software (1.54p, NIH, Bethesda, MD, USA). Because standard fluorescence microscopy (not confocal microscopy) was used, cells were washed three times with PBS (0.01 M, pH 7.4) prior to fixation to remove surface-associated DOX. For quantitative analysis, intracellular regions of interest (ROIs) were manually selected after applying a uniform threshold, while excluding membrane-localized and background signals. Mean fluorescence intensity was then calculated using ImageJ.

### 2.10. In Vivo Therapeutic Efficacy Test in Tumor Bearing Model

All animal procedures received approval from the Institutional Animal Care and Use Committee (IACUC) of Soonchunhyang University (approval number: SCH25-0004). Animals were housed in SPF barrier facilities under controlled environmental conditions (22 ± 2 °C, 50 ± 15% humidity, 12 h light/12 h dark cycle, and 13–16 air changes per hour) with unrestricted access to Teklad Global 18% Protein Rodent Diet (Envigo 2018, Indianapolis, IN, USA) and autoclaved water. Male BALB/c nude mice (5–6 weeks old) were subcutaneously injected at the dorsal flank with MDA-MB-231 cells (5 × 10^6^ cells in 200 μL, 0.01 M PBS). Upon tumor volume reaching approximately 50–100 mm^3^, animals were randomized into groups and intravenously administered PBS (0.01 M, pH 7.4), free DOX (10 mg/kg), or DOX-STs. Tumor size was measured with a digital caliper, and volume was calculated. Body weights were tracked daily, and mice were euthanized if their body weight decreased by more than 20% of the initial value. All animals were euthanized using CO_2_ inhalation in accordance with the approved IACUC protocol. For pharmacokinetic studies, blood samples were collected in heparinized centrifuge tubes, allowed to stand at room temperature for more than 30 min, and then centrifuged at 13,000 rpm for 15 min at 4 °C. Plasma was separated and stored at −20 °C until DOX concentration analysis.

### 2.11. DOX Plasma Concentration

Blood collection was performed via retro-orbital bleeding. Plasma was mixed with 100 μL of pH 8.8, 1.0 M Tris buffer solution, and extraction was carried out twice by adding 3 mL of chloroform/methanol (9/1, *v*/*v*) mixture with stirring for 3 min. Following centrifugation, the organic layer was collected and evaporated to dryness at 30 °C under nitrogen stream. The resulting dry residues were dissolved in mobile phase and the supernatant was introduced into the chromatograph. Separation was performed on a ZORBAX Eclipse XDB-C18 column (Agilent; 4.6 × 250 mm, 5 μm). The mobile phase, comprising methanol–water (with 0.1% formic acid anhydrous and 0.1% ammonia solution (25%), pH 3.0) at a 60:40 ratio, was delivered at a flow rate of 1.0 mL/min. The column was maintained at 35 °C. Excitation and emission wavelengths were set at 475 and 580 nm, respectively.

### 2.12. Statistical Analysis

Statistical analyses were conducted using Student’s *t*-test for comparisons between two groups, whereas one-way analysis of variance (ANOVA) with Bonferroni’s post hoc test was applied for comparisons involving three or more groups. A *p*-value of less than 0.05 was considered indicative of statistical significance.

## 3. Results and Discussion

### 3.1. Characterization of Sterosome

**Figure 1:**
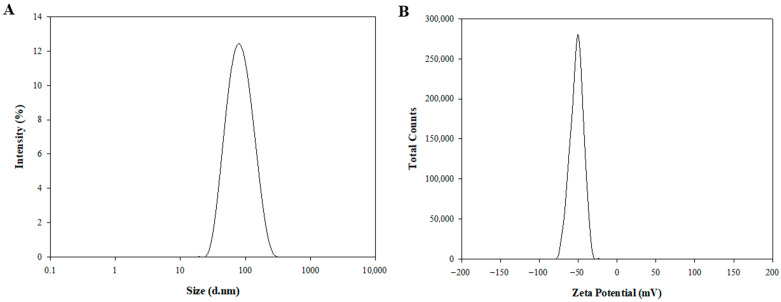
Physicochemical characterization of DOX-STs. (**A**) Particle size distribution and (**B**) zeta potential profiles of DOX-STs measured by DLS at pH 7.4. Data represent the mean ± SD from three independently prepared samples (*n* = 3).

In this study, sterosomes composed of non-phospholipid nanoparticles were prepared through self-assembly using the thin-film hydration technique. The physicochemical characteristics of the sterosomes are detailed in Table 1 and Figure 1. Dynamic light scattering (DLS) was used to assess the particle size, polydispersity index (PDI), and zeta potential. Blank-ST demonstrated an average diameter of 82.46 ± 0.73 nm, a PDI of 0.15 ± 0.01, and a zeta potential of −50.86 ± 0.48 mV (Table 1). PDI values around 0.15 indicate a narrow and uniform size distribution, reflecting excellent particle homogeneity. The pronounced negative zeta potential (approximately −50 mV) is mainly due to the deprotonation of PA. As the pKa of PA (~4.8) ensures that its carboxyl group remains ionized at physiological pH 7.4, PA is predominantly in its anionic form, imparting strong negative charges to the sterosome surface [46]. Such a high surface charge suggests significant electrostatic repulsion between particles, which helps prevent aggregation and contributes to improved long-term colloidal stability. Although nanoparticles with negative surface charge may be less efficiently internalized by cells compared to their positively charged counterparts, their reduced nonspecific interactions, prolonged systemic circulation, slower clearance, and lower cytotoxicity are advantageous for systemic delivery applications [47].

After encapsulation of DOX, the DOX-ST particle size was determined to be 80.73 ± 4.55 nm, with a PDI of 0.14 ± 0.01 (Figure 1A, Table 1), and a zeta potential of −53.09 ± 3.96 mV (Figure 1B, Table 1). There were no statistically significant differences in these parameters compared to Blank-ST, indicating that the sterosomes maintained their nanostructural integrity after drug encapsulation. As shown in TEM images (Figure S1), the sterosomes exhibited spherical and lamellar nanostructures with 3D architectures, measuring under 100 nm in diameter before and after DOX loading, consistent with the DLS results. This stability underscores the appropriateness of sterosomes as reliable nanocarriers for hydrophilic anticancer drugs such as DOX. In contrast to traditional phospholipid-based liposomes, which frequently display instability following drug loading, sterosomes preserved their physicochemical properties post-encapsulation. This result agrees with previous studies reporting that sterosomes possess enhanced structural durability and resistance to destabilization, emphasizing their potential benefits for drug delivery applications [22,46].

Furthermore, the drug loading content was 7.33%, and the encapsulation efficiency reached 93.95 ± 1.43% (Table 1). These data verify that DOX was encapsulated efficiently within the sterosomes. Achieving such high encapsulation efficiency may play a pivotal role in improving therapeutic outcomes by promoting effective drug delivery while minimizing adverse effects commonly seen with conventional drug formulations.

### 3.2. Sterosomes Stability

**Figure 2:**
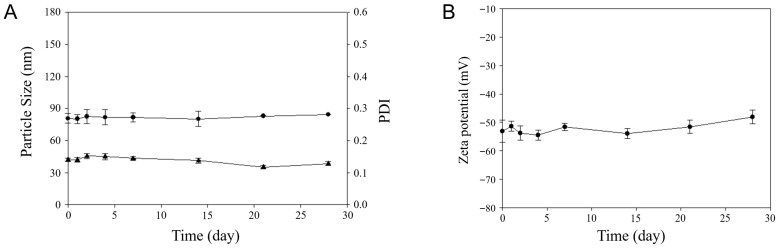
Stability evaluation of DOX-STs during storage. (**A**) Particle size (●) and PDI (▲), and (**B**) zeta potential of DOX-STs were measured over a 28-day period at 4 °C at pH 7.4. Data are presented as mean ± SD (*n* = 3).

For assessment of DOX-ST stability, particle size, PDI, and zeta potential were tracked during storage at 4 °C for 28 days using DLS. As depicted in Figure 2, the size, PDI, and zeta potential at day 28 measured 84.48 ± 0.02 nm, 0.13 ± 0.01, and −48.09 ± 2.39 mV, respectively, values not significantly different from their initial measurements (80.73 ± 0.73 nm, 0.14 ± 0.01, and −53.09 ± 3.96 mV on day 1). This confirms that the sterosomes retained their colloidal stability throughout the evaluation period. Stable and low PDI values (around 0.15) further confirm the narrow and uniform particle size distribution across the storage duration. Additionally, the zeta potential remained strongly negative (near −50 mV), indicating that electrostatic forces prevented particle aggregation over time. Altogether, these findings demonstrate that DOX-STs retain stable physicochemical characteristics during storage at 4 °C for at least 28 days.

**Figure 3:**
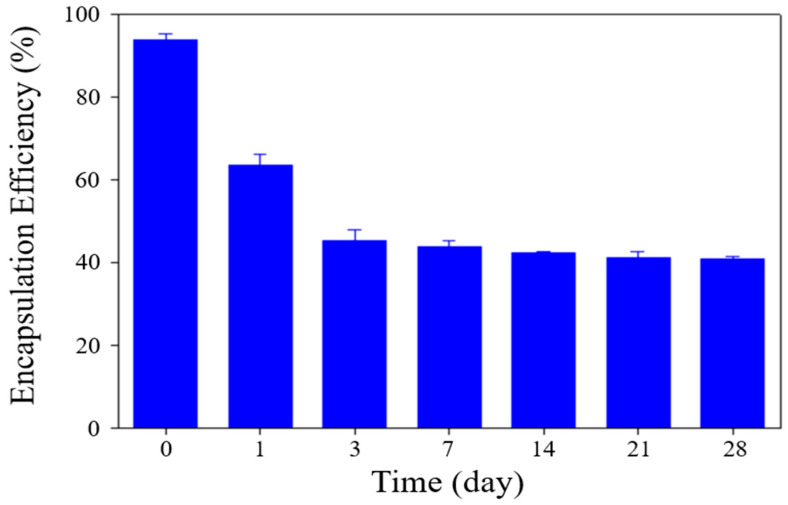
Encapsulation efficiency (EE) of DOX in sterosomes (DOX-STs) during storage. EE was continuously monitored over 28 days at 4 °C at pH 7.4 to assess the stability of the formulation. Data are presented as mean ± SD (*n* = 3).

Moreover, the EE of DOX within sterosomes was monitored during storage. As depicted in Figure 3, the EE experienced a substantial decline within the first three days, followed by a stabilization and maintenance of these values up to 28 days. This initial decrease aligns with previous reports by Chase S. Linsly et al. [45], who attributed this phenomenon to a thermodynamic stabilization process in lipid bilayers, which can lead to partial drug release during early storage, ultimately reducing encapsulation efficiency during the initial week. Based on these observations, it is reasonable to infer that a similar stabilization process was present in DOX-STs, causing early drug release, followed by subsequent equilibrium and retention of the remaining drug payload. This early decline in EE is most likely attributable to a transient lipid rearrangement process within the Chol/PA bilayer rather than uncontrolled leakage. As the bilayer transitions toward a thermodynamically stable packing state during the first 1–3 days, partial expulsion of loosely associated DOX molecules may occur [45]. Once this reorganization is complete, the sterosomes reach a stable structural configuration that minimizes further drug loss, consistent with the plateau observed after day 3. This mechanism aligns with previously reported behavior of sterol-containing vesicles undergoing early-phase membrane relaxation. Notably, after this initial adjustment period, the sterosomes maintained both their structural integrity and residual drug content, supporting their feasibility for short-term storage and potential clinical translation.

In addition to storage stability at 4 °C, we further assessed the physiological serum stability of DOX-STs by incubating the formulation in PBS (0.01 M, pH 7.4) containing 10% FBS at 37 °C for 24 h. The sterosomes maintained their colloidal integrity, with particle size, PDI, and zeta potential changing only slightly from their initial values (85.32 ± 3.37 nm, 0.19 ± 0.05, −47.98 ± 5.12 mV) to those measured after incubation (91.11 ± 4.22 nm, 0.21 ± 0.06, −49.98 ± 6.01 mV). A comparable trend was observed in drug retention: although the encapsulation efficiency decreased from 94.20 ± 1.91% to 65.33 ± 5.67% during the 24-h incubation, the formulation preserved stable morphology and surface charge, indicating resistance to aggregation and protein-induced destabilization. These findings demonstrate that DOX-STs exhibit similar stability behavior in physiological serum and under storage conditions, supporting their robustness in biologically relevant environments.

### 3.3. In Vitro Drug Release

**Figure 4:**
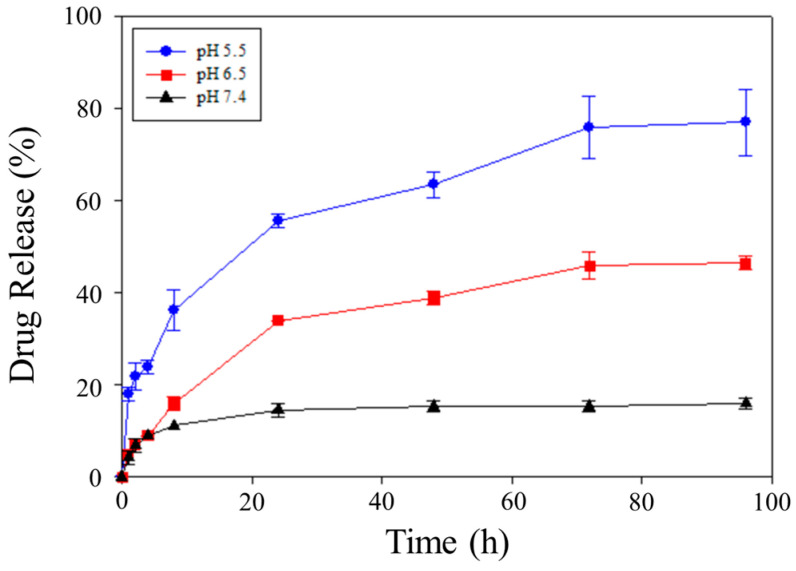
In vitro release kinetics of DOX from sterosomes (DOX-STs). Drug release experiments were conducted for 96 h in PBS (pH 7.4, 6.5, and 5.5) at 37 °C with gentle continuous agitation. All values are expressed as mean ± SD (*n* = 3).

In this study, the pH-responsive release of DOX from DOX-STs under varied pH conditions was examined utilizing the dialysis technique [48]. To simulate physiological and pathological conditions, PBS solutions (0.01 M, pH 7.4) at pH 7.4, 6.5, and 5.5 containing 1% Tween 80 served as release media, corresponding to blood, tumor, and lysosomal environments, respectively [48,49]. As shown in Figure 4, each pH condition displayed an initial burst release of DOX followed by a sustained release that was pH-dependent. At 96 h, cumulative DOX release reached 15.97% at pH 7.4, while notably greater release was attained under acidic pH, reaching 46.53% at pH 6.5 and 77.08% at pH 5.5. Notably, an initial burst release was observed within the first 4 h, amounting to approximately 6–8% at pH 7.4, 15–20% at pH 6.5, and nearly 30% at pH 5.5, before transitioning into a slower and sustained release phase. Collectively, these results underscore that DOX-STs facilitate enhanced release in acidic microenvironments compared with physiological pH. While a two-timescale kinetic model, as previously proposed for PLGA-based systems, could provide quantitative estimates of the burst and sustained release phases, such modeling is beyond the scope of this study and will be explored in future work [32].

This pH-dependent release profile results from the protonation of PA in acidic environments. At physiological pH (7.4), PA remains largely in its deprotonated carboxylate form (-COO^−^), which establishes robust electrostatic interactions and hydrogen bonding with Chol, thus ensuring the stability of the sterosome membrane. By contrast, at reduced pH levels, PA is present in its protonated form (-COOH), lessening electrostatic forces and diminishing hydrogen bonding, which promotes partial destabilization of the sterosome bilayer. As a consequence, this membrane destabilization increases the diffusion of DOX from the nanoparticles, consistent with earlier findings on PA/Chol systems [45,50]. In this context, the Chol/PA composition critically governs bilayer rigidity and the effective diffusion distance for DOX release, where cholesterol-induced membrane packing increases bilayer rigidity under physiological pH, whereas protonation of PA under acidic conditions reduces bilayer cohesion, shortens the diffusion barrier, and facilitates accelerated drug release. Unlike conventional phospholipid bilayers that exhibit sharp thermotropic phase transitions, the Chol/PA sterosome bilayer maintains a thermally stable and ordered structure over physiological temperature ranges due to cholesterol-mediated membrane packing, thereby enabling stable DOX encapsulation and rendering drug release predominantly governed by pH-induced bilayer destabilization rather than temperature-driven phase transitions. Compared with conventional phospholipid liposomes that undergo well-defined gel–liquid crystalline phase transitions accompanied by abrupt permeability changes, Chol/PA-based sterosomes exhibit cholesterol-stabilized bilayer packing with suppressed phase transitions and lower baseline permeability, thereby minimizing premature drug leakage while allowing release to be selectively triggered by chemical stimuli such as acidic pH.

In addition, the release pattern is influenced by the ionization state of DOX. When exposed to acidic pH, DOX undergoes protonation (-NH_3_^+^), which reduces its ability to form electrostatic interactions with protonated PA (-COOH). Therefore, the combined effects of membrane instability caused by PA protonation and reduced DOX–PA interaction are likely responsible for the notable burst release observed in acidic conditions. Alternatively, at a moderately acidic pH (6.5), partially maintained electrostatic interactions between deprotonated PA (-COO^−^) and protonated DOX, along with persistent hydrophobic interactions between anthraquinone rings of DOX and the hydrocarbon chains of PA, facilitated a more controlled and sustained release [51,52]. At physiological pH (7.4), sterosomes maintained a low level of DOX release, signifying high structural stability and persistent PA–DOX interactions in these conditions. This pH-dependent release behavior indicates that DOX-STs remain stable during systemic circulation, yet permit release to be triggered in acidic locations such as tumors or lysosomes. Collectively, these findings underscore the pH-responsive mechanism of DOX-STs, which offers two major advantages: (i) prolonged circulation stability that curtails premature drug leakage, and (ii) targeted DOX release in acidic tumor environments to reduce systemic toxicity and improve therapeutic outcomes.

### 3.4. In Vitro Cell Viability Assessment

Cytotoxicity of sterosomes was analyzed using the MTT assay on HepG2 (liver cancer), MDA-MB-231 (triple-negative breast cancer), A549 (lung cancer), and MCF-7 (breast cancer) cell lines. Blank-STs showed greater than 80% cell viability across all cell types (Figure S2), and additional evaluation in NIH 3T3 fibroblasts similarly demonstrated over 80% viability, confirming that unloaded sterosomes are biocompatible and do not markedly compromise cell viability. For DOX-STs, the indicated concentrations represent the actual encapsulated DOX (DOX-equivalent) concentration calculated from the measured encapsulation efficiency, ensuring a direct dose-matched comparison with free DOX.

**Figure 5:**
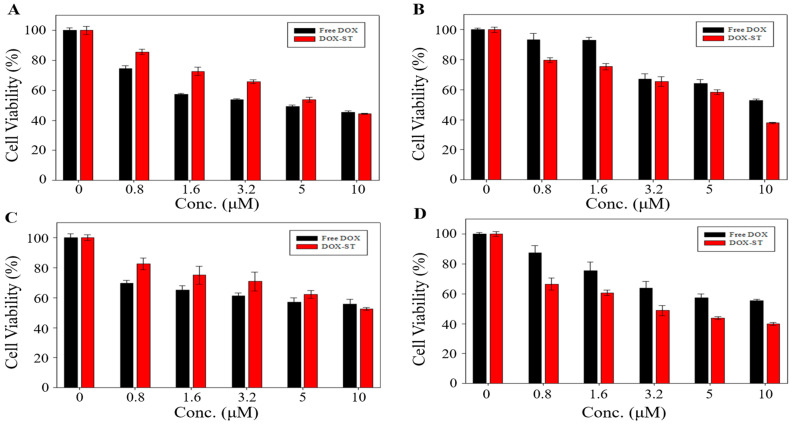
In vitro cytotoxicity of free DOX and DOX-STs. Cell viability was assessed using the MTT assay after 24 h incubation in (**A**) HepG2, (**B**) MDA-MB-231, (**C**) A549, and (**D**) MCF-7 human tumor cells. Results are expressed as mean ± SD (*n* = 4). For DOX-ST samples, the indicated concentrations correspond to the encapsulated DOX (DOX-equivalent) concentration, calculated based on the measured encapsulation efficiency.

Figure 5 presents the comparative cytotoxicity of DOX-STs and free DOX. In HepG2 and A549 cells, cytotoxicity did not differ significantly between free DOX and DOX-STs (45.49% vs. 44.42% for HepG2, Figure 5A; 55.67% vs. 52.52% for A549 at 10 μM, Figure 5C). In contrast, DOX-STs exhibited substantially greater cytotoxicity in MDA-MB-231 and MCF-7 cells, decreasing cell viability to 37.42% vs. 57.45% (MDA-MB-231, Figure 5B) and 39.85% vs. 55.44% (MCF-7, Figure 5D) at 10 μM, respectively. Correspondingly, IC_50_ values (Table 2) demonstrated that DOX-STs achieved lower IC_50_ values than free DOX in both breast cancer cell lines, suggesting increased efficacy at reduced concentrations.

The enhanced anticancer efficacy of DOX-STs in MDA-MB-231 and MCF-7 cells may arise from the synergistic effects of PA and Chol, the principal sterosome constituents. PA, which is a saturated long-chain fatty acid, modulates cancer cell proliferation and apoptosis. Multiple studies have shown that PA induces cell cycle arrest, upregulates pro-apoptotic markers (BAX, p53), and downregulates anti-apoptotic Bcl-2, thereby facilitating mitochondrial-dependent apoptosis [53,54,55,56,57,58,59]. However, the effects of PA are concentration-dependent: at low concentrations, PA does not markedly reduce cell viability but can inhibit cellular proliferation [53,60]. This finding is consistent with our observation that Blank-STs have minimal cytotoxicity while exhibiting stronger proliferation inhibition in breast cancer cells.

Taken together, these results indicate that PA does not exert direct cytotoxicity at the concentrations used in DOX-STs but instead provides a complementary pro-apoptotic stimulus that enhances the overall anticancer effect when combined with DOX. Therefore, PA acts as a synergistic modulator rather than an independent cytotoxic agent under our experimental conditions.

Furthermore, Chol is likely to enhance sterosome uptake by breast cancer cells through the very-low-density lipoprotein receptor (VLDLR), which is known to be overexpressed in breast cancer tissues and in cell lines such as MCF-7 and MDA-MB-231 [61,62]. Heightened VLDLR expression may increase the internalization of Chol-containing sterosomes, resulting in higher intracellular DOX delivery and consequently greater cytotoxicity than that observed with free DOX.

In contrast, HepG2 and A549 cells express substantially lower levels of VLDLR and exhibit reduced sensitivity to PA-mediated apoptotic signaling, which likely limits sterosome uptake and diminishes the synergistic enhancement observed in breast cancer cells.

Collectively, these data indicate that the enhanced cytotoxicity of DOX-STs in breast cancer cells results from: (i) PA-driven suppression of cell proliferation and induction of apoptosis, (ii) VLDLR-facilitated internalization of Chol-rich sterosomes, and (iii) pH-responsive DOX release in the tumor environment. In contrast, the absence of a notable increase in cytotoxicity in HepG2 and A549 cells highlights tumor-type-dependent differences in sterosome uptake and sensitivity.

### 3.5. In Vitro Cellular Uptake

**Figure 6:**
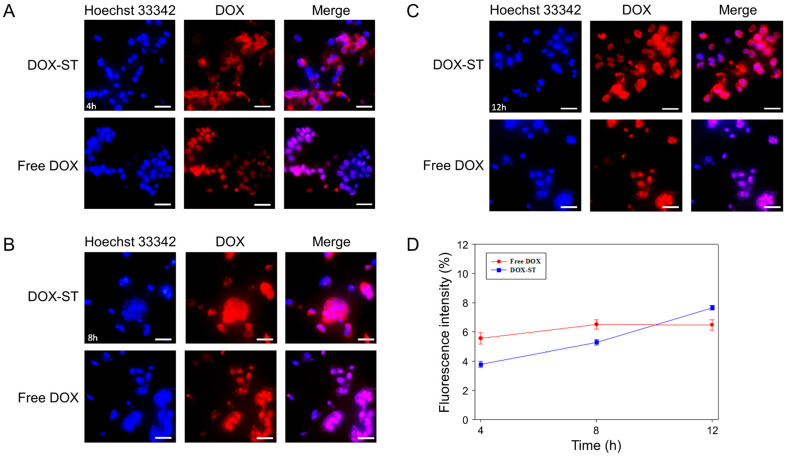
Cellular uptake of free DOX and DOX-STs in MCF-7 cells. Cells were treated with 5 μM of free DOX or DOX-STs, and intracellular localization was evaluated at multiple time points. (**A**) Fluorescence microscopy images at 4 h, (**B**) at 8 h, and (**C**) at 12 h. Nuclei were visualized using Hoechst 33342 (blue), and DOX fluorescence was detected in the red channel. Regions where blue and red signals overlapped appeared as a pinkish-purple merge. Scale bar = 50 μm. (**D**) Quantitative assessment of cellular uptake fluorescence intensity at 4, 8, and 12 h. Results are shown as mean ± SD (*n* = 3).

**Figure 7:**
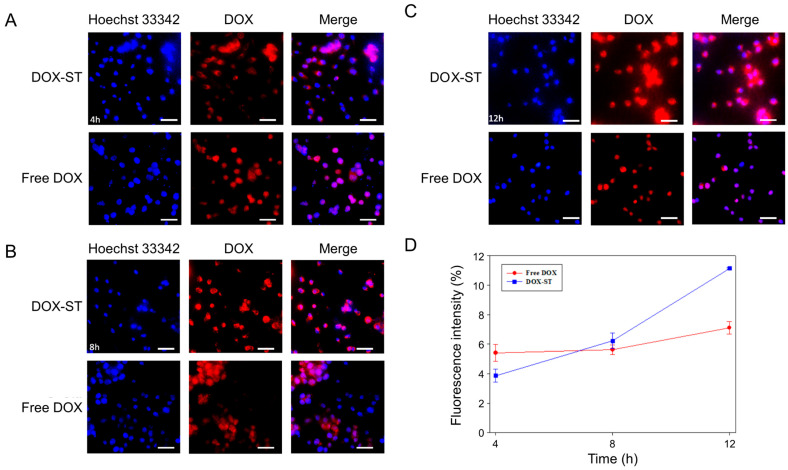
Cellular uptake of free DOX and DOX-STs in MDA-MB-231 cells. Cells were incubated with 5 μM of free DOX or DOX-STs, and the intracellular distribution was evaluated at different time intervals. (**A**) Fluorescence microscopy images after 4 h. (**B**) Images after 8 h. (**C**) Images after 12 h. Nuclei were visualized using Hoechst 33342 (blue), and DOX fluorescence was detected in the red channel. Regions where blue and red signals overlapped appeared as a pinkish-purple merge. Scale bar = 50 μm. (**D**) Quantification of fluorescence intensity demonstrating cellular uptake at 4, 8, and 12 h. Data are expressed as mean ± SD (*n* = 3).

Studies of cellular uptake were conducted to determine the time-dependent internalization of DOX-STs in MCF-7 and MDA-MB-231 human breast cancer cell lines. Analysis of fluorescence microscopy images and quantitative intensity measurements demonstrated significant differences in uptake characteristics between DOX-STs and free DOX (Figure 6 and Figure 7). In MCF-7 cells, DOX-STs showed lower fluorescence intensity at 4 and 8 h relative to free DOX (Figure 6A,B). By 12 h, however, DOX-STs resulted in greater nuclear accumulation compared with free DOX (Figure 6C). The increased colocalization of DOX fluorescence (red) with Hoechst-labeled nuclei (blue) indicates that DOX released from sterosomes effectively migrated to the nucleus, in agreement with the mechanism of DOX as a DNA intercalator [63]. Quantitative analysis (Figure 6D) supported this observation, revealing that DOX-STs surpassed free DOX in fluorescence intensity at the 12 h time point, indicative of a time-dependent pattern of intracellular uptake.

A parallel pattern was noted in MDA-MB-231 cells. At 4 h, the fluorescence from DOX-STs was weaker than that from free DOX (Figure 7A), but at 8 and 12 h, the intracellular DOX fluorescence from DOX-STs markedly increased (Figure 7B,C). Fluorescence intensity quantification (Figure 7D) demonstrated a substantial increase in cellular uptake with time, and DOX-STs ultimately exceeded free DOX levels. These findings indicate that sterosomes facilitate progressive internalization and controlled release of DOX, contributing to prolonged nuclear accumulation. The delayed yet enhanced nuclear localization of DOX observed for DOX-STs is kinetically consistent with endocytic uptake followed by pH-triggered drug release in acidic endosomal/lysosomal compartments, where sterosome destabilization facilitates DOX liberation, endosomal escape, and subsequent time-dependent nuclear translocation.

The increased intracellular uptake of DOX-STs can be attributed to two primary mechanisms: (i) PA metabolism in breast cancer cells. Seher Balaban et al. showed that β-oxidation of PA is diminished in MDA-MB-231 cells, favoring its incorporation into triacylglycerides (TAGs) [64]. This altered metabolism may increase the intracellular retention of PA-containing sterosomes, resulting in greater fluorescence intensity over time. (ii) VLDLR overexpression. Both MCF-7 and MDA-MB-231 cells demonstrate elevated levels of VLDLR [61,62], which promotes the uptake of Chol-rich nanoparticles. As sterosomes contain both PA and Chol, VLDLR-mediated endocytosis likely enhances their cellular uptake.

In addition to VLDLR-mediated uptake, previous studies have reported that sterol-rich vesicles and cholesterol-containing nanocarriers can enter cells through lipid raft-associated pathways, including caveolae-mediated and other clathrin-independent endocytic routes [65,66]. Cholesterol enrichment within the sterosome bilayer may promote interactions with plasma membrane microdomains, facilitating internalization through raft-dependent mechanisms [67,68]. Therefore, while VLDLR likely contributes to the uptake of Chol/PA-based sterosomes, additional non-receptor-specific, membrane domain-mediated pathways may also be involved [69]. Although pathway-specific inhibition experiments were not performed in this study, the observed uptake behavior and sterol-rich composition of the sterosomes support the involvement of multiple endocytic pathways rather than a single exclusive mechanism.

### 3.6. In Vivo Anti-Tumor Efficacy in Tumor Xenograft Model

To further investigate the therapeutic efficacy of sterosomes in breast cancer, an in vivo xenograft mouse model was established. Mice received intravenous injections of 8 mg/kg of free DOX or a dose-equivalent amount of DOX-STs, with PBS (0.01 M, pH 7.4) serving as the control. Tumor growth was evaluated every two days. Due to rapid tumor enlargement in the PBS group (surpassing 400% of baseline volume by day 16), all animals were euthanized at day 14.

**Figure 8:**
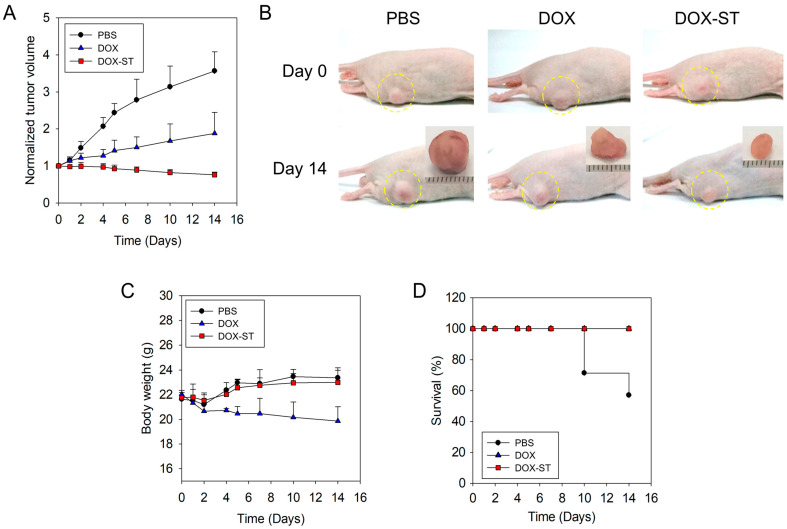
In vivo anti-tumor efficacy of DOX and DOX-STs in a breast cancer xenograft model. (**A**) Relative tumor volume, (**B**) representative tumor images collected on Day 0 and Day 14 after treatment, (**C**) relative body weight of mice, and (**D**) survival curves in different treatment groups (PBS, ●; DOX, ▲; DOX-ST, ■). Yellow circles indicate tumor regions. Data are presented as mean ± SEM (*n* = 5).

As shown in Figure 8A,B, the PBS group experienced a rapid fourfold increase in tumor volume from baseline. Free DOX resulted in moderate tumor growth suppression, while DOX-STs produced the most substantial inhibitory effect, significantly reducing tumor burden. These results support the superior anti-tumor activity of DOX-STs when compared with free DOX.

Body weight was monitored to evaluate systemic toxicity (Figure 8C). The PBS and DOX-ST groups maintained stable or slightly increasing body weights throughout the duration of the study, suggesting favorable tolerability. Conversely, mice administered free DOX displayed mild weight loss during treatment, indicative of systemic toxicity. Notably, the DOX-ST group exhibited no significant loss in body weight, further supporting its favorable safety profile.

Survival curves (Figure 8D) indicated that all mice in the DOX and DOX-ST groups remained alive throughout the experimental period, whereas mice in the PBS group experienced decreased survival, with a median endpoint of 14 days. These data indicate that DOX-STs not only enhance anti-tumor efficacy but also extend survival with minimal systemic toxicity.

**Figure 9:**
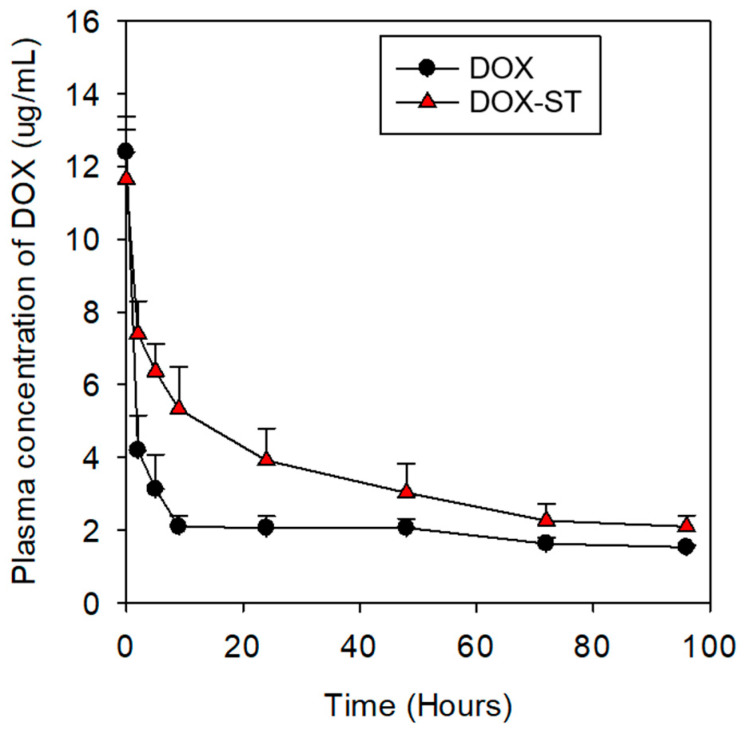
Plasma concentration–time profiles of DOX following intravenous administration. DOX levels in plasma were quantified at predetermined time points after intravenous injection of free DOX (●) or DOX-STs (▲) in a breast cancer xenograft mouse model. Data are presented as mean ± SEM (*n* = 3).

Furthermore, the plasma concentration–time profiles of DOX were evaluated by measuring plasma levels at eight predetermined time points following intravenous administration (Figure 9). DOX-STs exhibited higher and more sustained plasma concentrations than free DOX over the observed time course. At 1 and 2 h post-injection, plasma DOX levels of DOX-STs were approximately 2–2.5-fold higher than those of free DOX, with elevated concentrations persisting up to 96 h. These results suggest that sterosomes prolong the systemic presence of DOX in circulation, which may contribute to the improved therapeutic performance observed in vivo.

To further describe systemic exposure, non-compartmental pharmacokinetic parameters were estimated from the plasma concentration–time profiles following intravenous administration at a fixed dose (Table S1). The area under the curve (AUC_0–96_) was calculated using the trapezoidal rule, and systemic clearance (CL) was derived as CL = Dose/AUC_0–96_. In addition, the time required for plasma DOX concentration to decrease to 50% of the initial value (T50%) was estimated as a kinetic descriptor of early-to-intermediate circulation persistence. DOX-STs exhibited a higher AUC_0–96_, lower clearance, and markedly prolonged T50% compared with free DOX, indicating enhanced systemic exposure.

A separate Blank-ST group was not included in the in vivo experiments because both sterosome components, PA and Chol, are endogenous or widely used dietary lipids that exhibit minimal toxicity at physiologically relevant levels. This was further supported by our in vitro results, in which Blank-STs maintained >80% cell viability across all cancer cell lines and NIH3T3 fibroblasts (Figure S2). Given the negligible cytotoxicity and the absence of observable biological effects from empty sterosomes in vitro, we focused the in vivo evaluation on therapeutic performance rather than safety profiling of Blank-STs. Nonetheless, future studies incorporating an empty sterosome group may help further isolate nanoparticle-specific biological contributions.

Collectively, the in vivo findings validate that sterosomes offer both improved anti-tumor efficacy and decreased systemic toxicity, thereby enhancing the therapeutic efficacy and safety profile relative to free DOX.

Although sterosomes are composed of endogenous lipids such as Chol and PA, repeated administration of lipid-based nanocarriers can potentially trigger the accelerated blood clearance (ABC) phenomenon due to immune recognition. Previous studies have shown that repeated dosing of liposomes or sterol-rich vesicles may induce anti-PEG or anti-lipid IgM production, leading to rapid hepatic uptake and reduced circulation time during subsequent injections [70]. While sterosomes in this study were administered as a single dose, future investigations should assess whether repeated injections induce ABC-like responses, particularly regarding complement activation, IgM binding, and Kupffer cell-mediated clearance.

Compared with clinically used PEGylated liposomal formulations such as Doxil, which typically exhibit drug loading contents of ~1–5% and require complex PEGylation steps, our Chol/PA-based sterosomes achieve higher encapsulation efficiency (>90%) with a simplified lipid composition and standard thin-film hydration method [71,72]. The PEG-free design avoids potential PEG-associated immunogenicity and reduces manufacturing complexity, which may translate into lower production costs and improved translational feasibility [70]. Moreover, the pH-responsive release profile of DOX-STs offers a mechanistic advantage over conventional liposomes by enabling triggered drug release in acidic tumor microenvironments, potentially reducing off-target toxicity while maintaining therapeutic efficacy [73].

## 4. Conclusions

In this study, DOX-STs were systematically developed and comprehensively assessed for their physicochemical properties, in vitro performance, and in vivo efficacy. DOX-STs demonstrated high EE and maintained excellent storage stability for up to 28 days at 4 °C, supporting their practical applicability. In vitro release experiments demonstrated a pH-responsive release pattern, with more rapid drug release in acidic environments, simulating lysosomal and tumor microenvironments. Biological assessments further established the therapeutic advantages of DOX-STs. Blank-STs were found to be biocompatible, while DOX-STs markedly decreased cancer cell viability, especially in breast cancer cell lines (MDA-MB-231 and MCF-7), where they surpassed free DOX. Cellular uptake analysis revealed time-dependent internalization and increased nuclear localization of DOX-STs, aligning with their enhanced cytotoxicity in these cell models. In vivo, DOX-STs provided significant tumor growth suppression in a breast cancer xenograft model and reduced systemic toxicity, as evidenced by stable body weight and extended survival compared to free DOX. Pharmacokinetic evaluations showed that DOX-STs resulted in sustained plasma DOX levels and prolonged circulation, contributing to greater systemic exposure and improved therapeutic outcomes. Collectively, these results suggest that sterosome-based delivery offers a promising strategy for DOX administration by supporting long-term stability, regulated release, enhanced cellular internalization, and superior in vivo antitumor efficacy. Notably, the capacity of DOX-STs to reduce systemic toxicity while improving efficacy highlights their potential as a clinically relevant nanocarrier platform for breast cancer treatment.

## Figures and Tables

**Table 1:** Size, zeta potential, PDI, EE, and DLC of Blank-ST and DOX-ST, respectively. Data are presented as mean ± SD (*n* = 3).

	Size (nm)	Zeta Potential (mV)	PDI	EE (%)	DLC (%)
Blank-ST	82.46 ± 0.73	−50.86 ± 0.48	0.15 ±0.01	—	—
DOX-ST	80.73 ± 4.55	−53.09 ± 3.96	0.14 ± 0.01	93.95 ± 1.42	7.33

**Table 2:** IC_50_ values of Blank-STs, Free DOX, and DOX-STs against HepG2, MDA-MB-231, A549, and MCF-7 cells. Data are expressed as mean (*n* = 4) ± SD.

	HepG2	MDA-MB-231	A549	MCF-7
Blank-ST	—	—	—	—
Free DOX (μM)	9.39 ± 0.62	13.78 ± 0.07	12.86 ± 1.01	14.85 ± 0.11
DOX-ST (μM)	11 ± 0.44	9.85 ± 0.02	14.23 ± 0.27	7.66 ± 0.07

## Data Availability

Dataset available on request from the authors.

## Supplementary Materials

The following supporting information can be downloaded at: https://www.mdpi.com/article/10.3390/pharmaceutics17121574/s1, Figure S1: Representative TEM images of (A) Blank-STs and (B) DOX-STs. Figure S2: In vitro cytotoxicity of Blank-STs. Table S1. Non-compartmental pharmacokinetic parameters estimated from plasma concentration–time profiles of DOX following intravenous administration of free DOX or DOX-STs.
