# Supplementary material for: Stable Cholesterol–Palmitic Acid Sterosomes as Smart Nanocarriers for pH-Sensitive Doxorubicin Delivery in Breast Cancer Therapy
Source: Pharmaceutics. 2025 Dec 6;17(12):1574. doi: 10.3390/pharmaceutics17121574 (PMC12736873; doi:10.3390/pharmaceutics17121574)
Supplement: Supplementary file 1 [file pharmaceutics-17-01574-s001.zip › pharmaceutics-3986402-supplementary.pdf]

pharmaceutics

MDPI

Supplementary Materials: Stable Cholesterol–Palmitic Acid Sterosomes as Smart Nanocarriers for pH-Sensitive Doxorubicin Delivery in Breast Cancer Therapy

Jeong Min Lee [1,†], Chung-Sung Lee [2,3,†], Chae Yeong Lee [1], Min Lee [4,5,*] and Hee Sook Hwang [1,*]

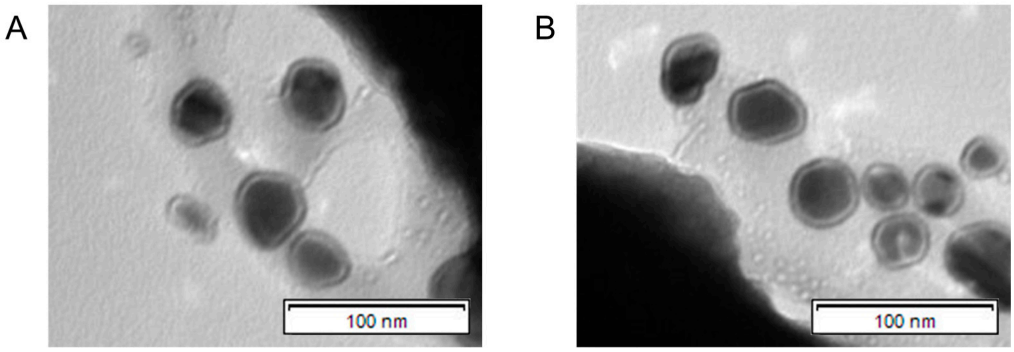

Figure S1. Representative TEM images of (A) Blank-STs and (B) DOX-STs present the morphology and relative size of the nanoparticles. Scale bars in TEM images represent 100 nm.

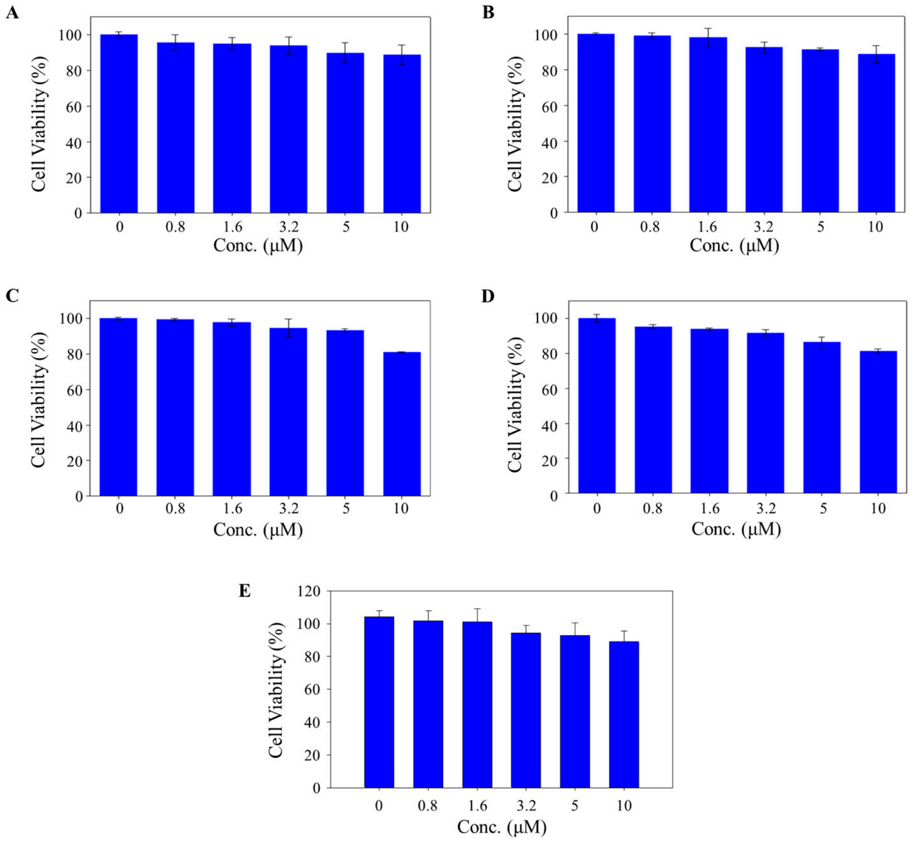

Figure S2. *In vitro* cytotoxicity of Blank-STs. Cell viability was assessed in (A) HepG2, (B) MDA-MB-231, (C) A549, (D) MCF-7 human tumor cells, and (E) NIH3T3 fibroblasts after 24 h incubation. Data are presented as mean ± SD (n = 4).

 https://doi.org/10.3390/pharmaceutics17121574

Table S1. Non-compartmental pharmacokinetic parameters estimated from plasma concentration–time profiles of DOX following intravenous administration of free DOX or DOX-STs.

Parameter	Free DOX	DOX-STs
$AUC_{0–96}$ (μg·h/mL)	201.3	331.9
CL (mL/h/kg)	49.7	30.1
T50% (h)*	1.5	7.1

* T50%: time required for the plasma DOX concentration to decreas.
